# Impact of Matching Point Selections on Image Registration Accuracy between Optical Scan and Computed Tomography

**DOI:** 10.1155/2020/3285431

**Published:** 2020-08-04

**Authors:** Hai Yen Mai, Du-Hyeong Lee

**Affiliations:** ^1^Department of Prosthodontics, School of Dentistry, Kyungpook National University, 2177 Dalgubeoldae-ro, Jung-Gu, Daegu 41940, Republic of Korea; ^2^Institute for Translational Research in Dentistry, Kyungpook National University, Daegu 41940, Republic of Korea

## Abstract

The point-based surface registration method involves the manual selection process of paired matching points on the data of computed tomography and optical scan. The purpose of this study was to investigate the impact of selection error and distribution of fiducial points on the accuracy of image matching between 3-dimensional (3D) images in dental planning software programs. Computed tomography and optical scan images of a partial edentulous dental arch were obtained. Image registration of the optical scan image to computed tomography was performed using the point-based surface registration method in planning software programs under different conditions of 3 fiducial points: point selection error (0, 1, or 2 mm), point distribution (unilateral, bilateral), and planning software (Implant Studio, Blue Bio Plan) (*n* = 5 per condition, *N* = 60). The accuracy of image registration at each condition was evaluated by measuring linear discrepancies between matched images at *X*, *Y*, and *Z* axes. Kruskal-Wallis test, Mann-Whitney *U* test with Bonferroni correction, and 3-way analysis of variance were used to statistically analyse the measurement data (*α* = 0.05). No statistically significant difference was exhibited between the 0 and 1 mm point mismatch conditions in either unilateral or bilateral point distributions. The discrepancy values in the 2 mm mismatch condition were significantly different from the other mismatch conditions, especially in the unilateral point distribution (*P* < 0.05). Strong interactions among point selection error, distribution, and software programs on the image registration were found (*P* < 0.001). Minor matching point selection error did not influence the accuracy of point-based automatic image registration in the software programs. When the fiducial points are distributed unilaterally with large point selection error, the image matching accuracy could be decreased.

## 1. Introduction

Three-dimensional (3D) imaging technologies have enhanced the diagnostic modalities and treatment planning for implant, maxillofacial surgery, and orthodontic fields [1, 2]. Cone-beam computed tomography (CBCT) is representative, and the 3D image data that it produces are commonly used to evaluate the underlying bone and identifying critical anatomical structures, such as the inferior alveolar nerve, the maxillary sinus, and the roots of neighbouring teeth [3]. Slices of CBCT radiographic images can be reconstructed into a 3D image model [4], but the resolution of the 3D image is limited because of the voxel size of raw radiographic data available in CBCT devices [5, 6]. Thus, to make a 3D model with soft and hard tissue, image merging with optical scan data of the oral cavity surface is recommended [7, 8].

Image registration is the process of matching the optical scan image to the 3D-reconstructed CBCT image [9, 10]. Accurate image registration is essential to replicate the exact relationship of underlying bone and oral cavity surface data [11, 12]. Errors in the image alignment in this step could cause unexpected surgical complications because of discrepancies between the planning in the virtual model and the actual results at the surgical site [13]. Contemporary image registration techniques for 3D data are divided into voxel-based and surface-based method [14]. The voxel based-registration utilizes the grey-scale difference of the voxels to align the images, achieving the least difference in the total grey-scale density [14]. Because the voxel-based registration uses the image intensity in the entire volume, the presence of image artifacts caused by metallic prostheses in the radiographic data could deteriorate precise image matching by masking the fiducial anatomic structures [5]. Meanwhile, the surface-based registration uses the geometric shape of 3D object surfaces to match images [15]. The computer algorithm of iterative closest points (ICPs) locates optimal positions of pair images by computing the 3D coordinates of image surface point clouds [14]. The accuracy of image superimposition by graphic processing has been validated [16], and currently, point-based surface matching is widely used in the dental planning and inspection computer software programs for the enhancement of efficiency and accuracy of matching [9].

When performing the point-based surface registration, the congruent areas between the 3D reconstructed radiographic image and optical scan image are designated in pair [17]. In the dentate jaws, because the tooth is discernible in both images, the outline of the tooth is generally used as the fiducial area of image superimposition [17]. Three pair points have been used for image matching [18], and wide distribution of points in the arch is recommended [19]. It was also suggested to choose the matching points close to the edentulous area [19, 20]. Although several protocols for image registration have been suggested, the paired points could be selected differently because this process basically depends on the operator's visual observations. Human error in selecting matching points could affect the accuracy of subsequent automatic image matching processes. The recommended positions of the matching points also have not been clarified. The purpose of this study was to assess the impact of selection error and distribution of fiducial points on the accuracy of image matching between 3D radiographic and optical scan images in planning software programs. The proposed null hypothesis was that the mismatch in paired fiducial points, and the different distribution of points would not influence the accuracy of image registration of the 3D optical scan image to the reconstructed radiographic data.

## 2. Materials and Methods

**Figure 1:**
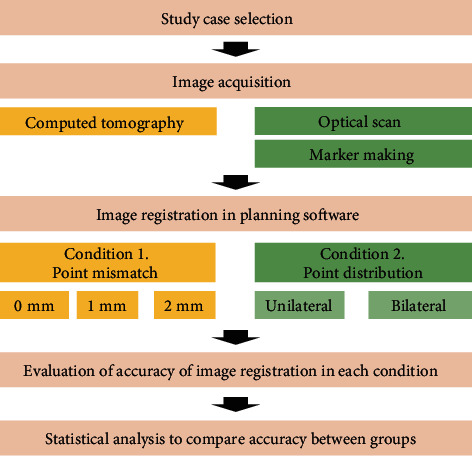
Workflow of this study.

**Figure 2:**
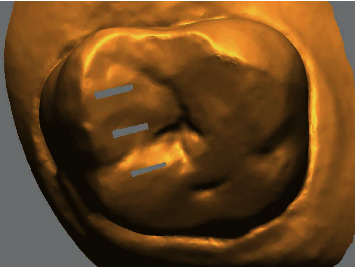
Indexing markers at intervals of 1 mm to guide the operator in placing matching points at different levels of error selection.

The workflow of this study is described in Figure 1. A patient missing the second premolar and first molar on the maxilla was selected for this study. The patient had no severe defect or metal restorations in the remaining teeth on the arch. Computed tomographic images of the patient were obtained by using a CBCT scanner (Pax-i3D Smart, Vatech, Hwaseong, Korea) with 80 kVp, 8 mA, 24 s pulsed scan, field of view of 100 × 80 mm, and slice thickness of 0.2 mm. An optical scan image of the oral cavity surface was obtained by digitizing the stone model using a lab-based scanner (IDC S1, Amann Girrbach, Koblach, Austria). The stone model was fabricated using the conventional silicone impression and stone pouring technique. The scan file was transferred to a reverse engineering software program (Geomagic Design X, 3D Systems, Rock Hill, SC, USA) where indexing markers for indicating matching points were made on the occlusal surface of teeth by deleting the surface of the image in the strip shape at 1 mm intervals (Figure 2). Consequently, the radiographic and optical-scan data were delivered to planning computer software programs, Implant Studio (3Shape, Copenhagen, Denmark) and Blue Sky Plan (Blue Sky Bio, LLC, Grayslake, IL, USA), for merging the images.

**Figure 3:**
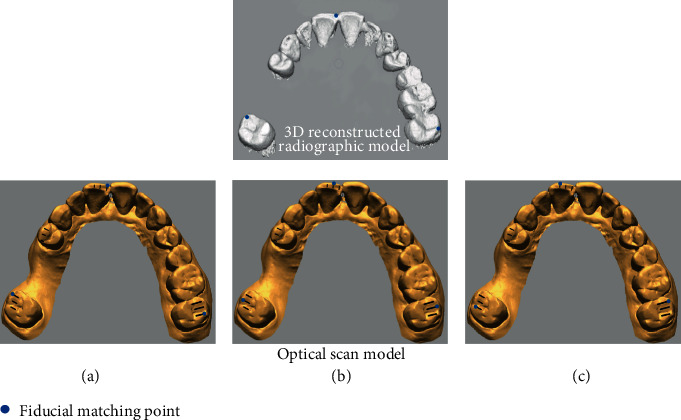
Mismatch conditioning of paired matching points between radiographic and optical scan images: (a) no mismatch, (b) 1 mm mismatch, and (c) 2 mm mismatch.

**Figure 4:**
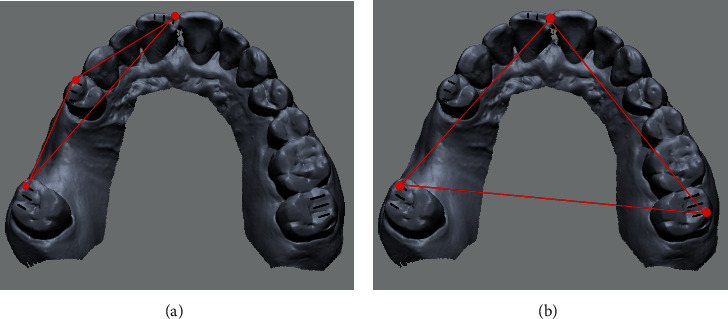
Conditioning of distribution of matching points: (a) unilateral and (b) bilateral.

The experimental factors of this study were selection error in pair points and the distribution of fiducial points in the image registration process. The selection error was set at 3 levels (0, 1, and 2 mm; Figure 3), and the distribution of points was set at 2 levels (unilateral and bilateral; Figure 4). In all matching conditions, 3 matching points were used in pairs in both radiographic and optical-scan images, and the positions of points were designated in reference to the indexing markers to provide the standardized error in point selection [17, 20]. The central incisor, first premolar, and second premolars were used for the image matching in the unilateral and bilateral distribution conditions. After the point designation in each condition, the point-based best-fit algorithm was run in the software programs. All image registrations were carried out 5 times in each condition by a single operator who had experience in image registration and was blinded to the purpose of this study.

**Figure 5:**
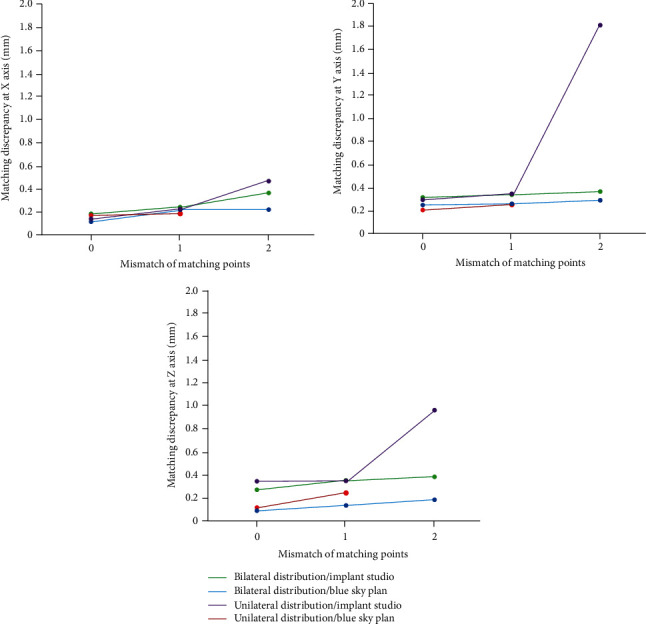
Line graphs showing the effect of the mismatch of matching points on the accuracy of image registration in different point distribution and software programs at *X*-, *Y*-, and *Z*-axes.

The resulting accuracy of each condition's image registration was evaluated by measuring the positional discrepancy between the radiographic and optical-scan images in the *X*-, *Y*-, and *Z*-axes (Figure 5). The assessments were performed in the cross-sectional images of central incisor and second molar areas using the measurement function of the planning software programs. A single investigator carried out all the measurements to avoid errors that can arise when using different examiners.

The mean and standard deviation of linear discrepancies in each condition were calculated by averaging the measurement values collected in the anterior and posterior areas. Kruskal-Wallis test and a post hoc Mann-Whitney *U* test with Bonferroni correction were used to compare the accuracy of image registration between groups. Three-way analysis of variance (ANOVA) was used to investigate how interactions between factors, such as the point selection error, point distribution, and planning software, affected image registration accuracy. All statistical analyses were performed by using the Statistical Package for the Social Sciences (SPSS) software program (SPSS version 25.0; IBM Inc., Armonk, NY, USA) with the statistical significance level of 0.05.

## 3. Results

The linear discrepancy between the radiographic and optical-scan images after the image registration process at different matching conditions is shown in Table 1. In general, no statistically significant difference was found between the 0 and 1 mm point mismatch conditions; however, the discrepancy values in the 2 mm mismatch condition were significantly different from the other mismatch conditions. In particular, when the 2 mm point mismatch condition was applied in the unilateral point distribution in the Blue Sky Plan software, markedly high discrepancy was observed (9.92 mm in the *X*-axis, 2.52 mm in the *Y*-axis, and 4.19 mm in the *Z*-axis).

The pattern of point distribution did not make a statistically significant difference in the matching discrepancy in the 0 and 1 mm point mismatch conditions, but it did make significant differences in the 2 mm mismatch condition in both planning software programs.

Three-way ANOVA results showed that each factor has strong interactions with different factors, which affects the outcome of image matching, as shown in Table 2 (*P* < 0.001; adjusted *R*^2^ = 0.958).

## 4. Discussion

The purpose of this study was to elucidate the impact of the matching point selection process on the accuracy of optical scan image registration to radiographic images for image merging. Variations in the process of manual point designation were designed by controlling the factors, such as the disagreement in paired points and the distribution of points. The overall results of this study showed that minor errors in matching point selection did not influence the accuracy of image registration regardless of whether the distribution pattern was unilateral or bilateral. However, the discrepancy was significantly different when there was large point mismatch. Thus, the null hypothesis that the mismatch in paired fiducial points and the different distribution of points would not influence the accuracy of image registration was rejected.

The image matching method used in the study was point-based surface registration. Polygonal 3D mesh images were aligned with each other in the closest position using the best-fit algorithm based on the fiducial points designated by the operator [21]. The results of this study showed that minor errors in the point selection did not affect the quality of image superimposition of paired images. This accurate matching algorithm not only reduces the influence of iatrogenic mistakes in the image superimposition for treatment planning but also can be used for result analyses and research purposes in various medical fields [22, 23]. Because the matching method is based on mathematical calculations, it is assumed that there is no matching error when the matching is performed in ideal conditions. However, in the present study, there was some discrepancy when there was no point selection error. This phenomenon might be due to the differences in forms of 3D restructured radiographic and optical scan images. Shape deformation has been reported in the conversion process from radiographic raw data to 3D mesh image because of the partial volume effect [24–26]. The optical scan model could also have dimensional errors depending on scanner performance and scanning strategy [27, 28]. Thus, operators should be aware of the errors that inevitably occur because of the morphological differences between matched images, and they should focus on minimizing additional operator errors caused by inappropriate manual works.

In this study, point-based surface registration was performed in planning software programs that are widely used in guided implant surgery [9]. The general results were similar in the 2 software programs. However, the matching results were different in unilateral point distribution with 2 mm selection error. In the Implant Studio, the optical scan image was approximately located near the corresponding radiographic image, but in the Blue Sky Plan, the image matching failed completely. The malfunctioning of the best-fit algorithm might be due to the fact that the level of point mismatch in a certain point distribution was beyond the condition that is needed for the automatic image matching to operate normally. Based on the findings of the present study, it seems that the maximum allowance limit that enables normal operation of best-fit image matching might be different according to different computer software programs. Scherer [23] reported that the accuracy of outcomes could vary depending on the software programs used. Further studies on the capability of image matching in different commercial software programs are needed.

The conventional procedures of silicone impression taking and gypsum cast fabrication in this study could be sources of error in replicating the morphology of the oral cavity because of the physical and chemical characteristics of the materials [29]. Intraoral optical scanning can be considered to directly obtain image data from the oral cavity, eliminating the drawbacks of analog methods [30]. A partially edentulous dental arch case was chosen for this study. Considering the clinical variability in the size of dental arches, positions and spans of the edentulous area, and tooth morphology, various clinical cases should be included in future studies to generalize the results of the present study. Cases with metallic restorations also need to be included to investigate the effects of metal artifact images on the accuracy of image registration. The proficiency of the operator in the image matching could be another influencing factor. The operator factor was controlled in the present study by recruiting operators who had the same amount of experience in using planning computer software. Clinically, given that operator's experience varies, it will be necessary to examine the relationship between experience and point selection error in related future studies.

## 5. Conclusions

Within the limitations of this study, the following conclusions were drawn:

1. A matching point selection error of 1 mm did not affect the accuracy of optical scan image registration to radiographic image in either unilateral or bilateral point distributions
2. Whether the fiducial points were distributed unilaterally or bilaterally did not affect the accuracy of image registration when there was no or 1 mm point selection error
3. The accuracy of image registration was significantly different between software programs when the fiducial points were distributed unilaterally with a 2 mm selection error

## Figures and Tables

**Table 1:** Mean and standard deviation of linear discrepancy of image matching at each condition (mm).

Coordinate	Distribution	Implant studio			*P*	Blue Sky Plan			*P*
		Point mismatch				Point mismatch			
		0	1	2		0	1	2	
*X*	Bilateral	0.20 ± 0.03^a^	0.22 ± 0.02^ab^	0.24 ± 0.03^b^	0.101	0.12 ± 0.07^a^	0.21 ± 0.08^ab^	0.21 ± 0.11^b^	0.055
	Unilateral	0.18 ± 0.02^a^	0.21 ± 0.02^a^	0.46 ± 0.14^b^	<0.001	0.18 ± 0.06^a^	0.19 ± 0.04^a^	9.92 ± 4.43^b^	<0.001
	*P*	0.205	.141	.006		0.096	.543	<0.001	
*Y*	Bilateral	0.31 ± 0.05	0.33 ± 0.07	0.37 ± 0.13	0.258	0.24 ± 0.17	0.25 ± 0.18	0.27 ± 0.32	0.901
	Unilateral	0.29 ± 0.08^a^	0.34 ± 0.10^a^	1.79 ± 0.21^b^	<0.001	0.20 ± 0.19^a^	0.24 ± 0.20^a^	2.52 ± 1.43^b^	<0.001
	*P*	0.951	0.651	<0.001		0.936	0.689	<0.001	
*Z*	Bilateral	0.30 ± 0.04^a^	0.35 ± 0.02^b^	0.39 ± 0.03^b^	<0.001	0.11 ± 0.02^a^	0.14 ± 0.04^b^	0.19 ± 0.01^c^	<0.001
	Unilateral	0.34 ± 0.04^a^	0.35 ± 0.02^a^	0.96 ± 0.47^b^	0.002	0.12 ± 0.02^a^	0.22 ± 0.03^a^	4.19 ± 2.54^b^	<0.001
	*P*	0.067	0.576	0.036		0.090	0.052	<0.001	

Values with the same letter are not statistically different based on Kruskal-Wallis test at *P* < 0.05.

**Table 2:** Variations between different affecting factors and interactions in the accuracy of image registration by 3-way analysis of variance.

Source	Type III sum of squares	d.f.	Mean square	*F*	*P*
Corrected model	1142.827	11	103.893	370.319	<0.001
Intercept	209.693	1	209.693	747.433	<0.001
Distribution	123.936	1	123.936	441.758	<0.001
Software	76.649	1	76.649	273.210	<0.001
Mismatch	257.151	2	128.576	458.297	<0.001
Mismatch × distribution	241.687	2	120.844	430.737	<0.001
Mismatch × software	174.877	2	87.439	311.668	<0.001
Distribution × software	90.653	1	90.653	323.124	<0.001
Mismatch × distribution × software	177.873	2	88.937	317.007	<0.001
Error	47.133	168	0.281		
Total	1399.652	180			
Corrected Total	1189.959	179			

Adjusted *R*^2^ = 0.958.

## Data Availability

The data used to support the findings of this study are included within the article.
